# Plant Growth Promotion and Suppression of Bacterial Leaf Blight in Rice by Inoculated Bacteria

**DOI:** 10.1371/journal.pone.0160688

**Published:** 2016-08-17

**Authors:** Sumera Yasmin, Abha Zaka, Asma Imran, Muhammad Awais Zahid, Sumaira Yousaf, Ghulam Rasul, Muhammad Arif, Muhammad Sajjad Mirza

**Affiliations:** 1 National Institute for Biotechnology and Genetic Engineering (NIBGE), Faisalabad, Pakistan; 2 Department of Plant Pathology, University College of Agriculture, University of Sargodha, Sargodha, Pakistan; 3 Nuclear Institute for Agriculture and Biology (NIAB), Faisalabad, Pakistan; Fujian Agriculture and Forestry University, CHINA

## Abstract

The present study was conducted to evaluate the potential of rice rhizosphere associated antagonistic bacteria for growth promotion and disease suppression of bacterial leaf blight (BLB). A total of 811 rhizospheric bacteria were isolated and screened against 3 prevalent strains of BLB pathogen *Xanthomonas oryzae* pv. *oryzae* (Xoo) of which five antagonistic bacteria, *i*.*e*., *Pseudomonas* spp. E227, E233, Rh323, *Serratia* sp. Rh269 and *Bacillus* sp. Rh219 showed antagonistic potential (zone of inhibition 1–19 mm). Production of siderophores was found to be the common biocontrol determinant and all the strains solubilized inorganic phosphate (82–116 μg mL^-1^) and produced indole acetic acid (0.48–1.85 mg L^-1^) *in vitro*. All antagonistic bacteria were non-pathogenic to rice, and their co-inoculation significantly improved plant health in terms of reduced diseased leaf area (80%), improved shoot length (31%), root length (41%) and plant dry weight (60%) as compared to infected control plants. Furthermore, under pathogen pressure, bacterial inoculation resulted in increased activity of defense related enzymes including phenylalanine ammonia-lyase and polyphenol oxidase, along with 86% increase in peroxidase and 53% increase in catalase enzyme activities in plants inoculated with *Pseudomonas* sp. Rh323 as well as co-inoculated plants. Bacterial strains showed good colonization potential in the rice rhizosphere up to 21 days after seed inoculation. Application of bacterial consortia in the field resulted in an increase of 31% in grain yield and 10% in straw yield over non-inoculated plots. Although, yield increase was statistically non-significant but was accomplished with overall saving of 20% chemical fertilizers. The study showed that *Pseudomonas* sp. Rh323 can be used to develop dual-purpose inoculum which can serve not only to suppress BLB but also to promote plant growth in rice.

## Introduction

Rice is among the oldest crops of the world which feeds about half of the world’s population [1]. In Pakistan rice is second major crop after wheat and main source of foreign exchange for the country after cotton. Of 70 different microbial diseases, bacterial leaf blight (BLB) is the most devastating in terms of annual yield losses to rice across the globe [2]. Different chemical and cultural approaches have been used for the management of BLB but results are not very encouraging or effective due to variability of pathogen, lack of durable resistance, insensitivity to different antibiotics or many other environmental factors [3].

Biological control of BLB using plant growth promoting rhizobacteria (PGPR) has emerged as an effective strategy during last two decades [4]. PGPR (either rhizospheric, rhizoplanic or endophytic) are plant root associated beneficial bacteria [5] that confer plant growth promotion [6]. The mechanisms of plant growth promotion by PGPR include production of growth hormones e.g., indole acetic acid (IAA), solubilization of inorganic phosphate [7; 8], fixation of atmospheric nitrogen, ACC deaminase activity [9] and zinc solubilization [10]. They can also be involved in promoting plant health by suppressing phytopathogens using various mechanisms *i*.*e*. competition, production of siderophores, antagonism and induced systemic resistance [11]. PGPR-induced systemic resistance activates the plant’s latent defense that leads to the activation of multiple defense-related compounds/ enzymes at sites distant from the pathogen attack [12]. Peroxidase (POD; EC 1.11.1.7) is one of the fast responding defense related enzymes against plant pathogens which are involved in lignification, suberification, polymerization of hydroxyproline-rich glycoproteins, regulation of cell wall elongation, wound healing, and resistance against pathogens in plants [13; 14; 15]. Catalase (CAT) is an oxygen-scavenging enzyme that protects cells from the toxic effects of H_2_O_2_ during development by converting it to water and molecular oxygen [16; 17; 18]. Polyphenol oxidase (PPO; EC1.10.3.1) is important in the initial stage of plant defense where membrane damage causes release of phenols such as chlorogenic acid. PPO catalyzes the oxidation of phenolics to free radicals that can react with biological molecules, thus creating an unfavorable environment for pathogen development [19]. Phenylalanine ammonia-lyase (PAL) is the primary enzyme in the phenyl propanoid metabolism and plays a significant role in the synthesis of several defense-related secondary compounds such as phenols and lignin [20; 21].

In Pakistan, BLB disease was first reported during the year 1977 and since then epidemically damaged the crop (80–100%) in areas of northern and central Punjab [22] and has caused millions of tons of grain loss per annum [23]. Incidence of BLB has increased up to many folds in the recent years especially in the Kallar belt under rice cultivation. BLB disease was considered as the main yield limiting factor in Basmati rice growing areas in 2013–2014 (Economic Survey of Pakistan, 2014). Basmati rice, being well known for its aroma and taste is specific to Indian sub-continent. Pakistan ranks 2^nd^ in terms of Basmati rice and 3^rd^ in total rice export in the world. At present, rice production mostly depends on synthetic fertilizers and pesticides which render un-healthy environment and polluted ecosystem in rice-producing areas [24]. Keeping in view the importance of growing quality rice, specifically ‘Basmati’ in the country in more environment-friendly way, this study was planned to select native antagonistic bacteria with the purpose to control BLB pathogen i.e., *Xanthomonas oryzae* pv. *oryzae* (Xoo) and secondly, to use them for PGPR-assisted plant growth promotion in field. The use of integrated plant nutrient management systems improves crop yield, plays a vital role in the development of sustainable agricultural systems for crop production [25] and guarantees a healthy environment. Many reports have been published that describe either the use of PGPR for growth promotion or the antagonistic bacteria to control pathogens but rice inoculum that can be used both as biofertilizer as well as biopesticide is not available in Pakistan. We have described the use of a single “dual-purpose inoculum” based upon native antagonistic-PGPR that can promote rice growth on one hand and control pathogen attack on the other hand. This dual purpose inoculum may serve as rice supplement for sustainable rice growth in the country.

## Materials and Methods

### Isolation and pathogenicity test of *Xanthomonas oryzae* pv. *oryzae* (Xoo)

Healthy and BLB infected rice leaf samples were collected from 12 different sites of rice growing belt in Punjab province. No permits were required for collection of plant samples, which complied with all relevant regulations. In addition, the said studies did not involve the endangered or protected species. Selected sampling sites were the hot spot of BLB in the year. Prevalent strains of Xoo were isolated from BLB infected samples, collected from these sites in 2013 on Potato Sucrose Agar medium [26] medium (sucrose 10g, peptone 10g, agar 12g, distilled water 1L, pH 7.4). Pathovar of isolated Xoo strains (Xoo8, Xoo9, Xoo10) was determined using molecular marker J03 (5'-GCTCAGGTCAGGTGGCCTGG-3') [27] and their virulence was confirmed using pathogenicity test on rice plants grown in pots under net house conditions as already described [3]. Xoo4 was used as reference strain for pathovar detection. Xoo strains were inoculated to rice plant by clip inoculation method [28] and data regarding disease severity was measured in terms of % diseased leaf area (DLA) after 14 and 21 days of inoculation.

### Isolation, identification and *in vitro* screening of rhizobacteria against Xoo

Total bacterial population was determined from rhizosphere, rhizoplane and endorhizosphere of the rice plants collected from 12 different sites using serial dilution method [29] on Luria Bertani (LB) agar medium. For the isolation from endorhizosphere, 1 g of roots were washed with tap water, surface sterilized with 5% NaHOCl (sodium hypochlorite) for 1 minute followed by repeated washing with distilled autoclaved water. The sterilized roots were rolled on LB agar plates to determine the efficacy of surface sterilization. The sterilized roots were crushed in sterilized pestle mortar and then added to test tube containing 9 mL of 0.85% saline for preparing serial dilutions. Aliquots (100 μL) of selected dilutions were spread on LB agar plates separately. The plates were incubated at 30±2°C for 24–48 hours. Representative colonies were picked and sub-cultured to get pure culture. The bacterial isolates (811) were screened *in vitro* for their antagonistic activity against eight Xoo strains using diffusion plate assay [30] with sterile distilled water as control. Xoo8, Xoo9, Xoo10 were isolated in the present study while Xoo2, Xoo4, Xoo5, Xoo7 and Xoo11 were reference strains obtained from NIBGE Biotech Resource Centre (NBRC), Faisalabad. Bacterial antagonists showing consistent antagonistic activity in repeated plate assays were selected and characterized on the basis of cultural characteristics, Gram reaction, fluorescence emission and biochemical characterization *i*.*e*. KOH solubility and catalase test using standard methods [31].

Bacterial antagonists were identified by sequencing16S rRNA gene. The 16S rRNA gene was amplified using primer pair PA (5' AGACTTTGATCCTGCTCAG 3') and PH 5 (5' AAGGAGGTGATCCAGCCGCA 3') as described by [32]. Amplified PCR product (1.5Kb) of 16S rRNA gene was cleaned using QIAquick Gel Extraction Kit (QIAGEN Sciences, Maryland 20874, USA) and commercially sequenced by Macrogen, Inc. (Seoul, South Korea). Sequence data was aligned and compared with the available sequences of bacterial lineage in National Center for Biotechnology Information (NCBI) Genbank (http://www.ncbi.nlm.nih.gov/) using BLAST. Sequences were submitted to NCBI GenBank database and accession numbers were obtained.

### Characterization of rhizobacteria for biocontrol and growth promoting traits

Ability of bacterial antagonists to produce siderophores was detected using universal chrome azurol ‘S’ (CAS) assay [33]. Development of pink coloration around the bacterial colonies indicated the production of siderophpores. Non-siderophore producing bacterial strain StRh2 (Tariq et al. 2010) was used as negative control. Production of hydrogen cyanide (HCN) was determined on King’s B medium amended with glycine (4.4 g L^-1^) [34]. Starch hydrolysis was detected by streaking bacteria on LB agar plates with 2% starch that were incubated at 30±2°C for 48–72 hours and then overlaid with the layer of Lugol’s solution. Development of clear zone around bacterial colonies was an indication of starch hydrolysis [35]. Ability to produce different lytic enzymes *i*.*e*. proteases, chitinases and glucanases was detected using skimmed milk agar medium, colloidal chitin-conatianing agar and laminarine-containing agar medium, respectively [36; 37]. Detection of the genes involved in antibiotic production i.e., phenazine, 2,4-diacetylphloroglucinol (2,4-DAPG), pyrrolnitrin was carried out by polymerase chain reaction [38]. The primers used and the PCR amplification conditions are provided as S1 Table.

IAA production was detected using spot test [39] and quantified through HPLC as described by [40]. Phosphate solubilization was detected by spotting bacteria on to Pikoviskaya’s agar plates containing tricalcium phosphate [41] and quantified using Phosphomolybdate blue color method [42]. Organic acids were extracted and analyzed using HPLC as reported by [43].

### Plant inoculation assays

#### Experiment I: Effect of bacterial antagonists on seed germination

Effect of antagonistic bacteria on germination of rice seeds (variety Super Basmati) was tested in a growth room experiment. Seeds were surface sterilized with 1% NaHOCl for 5 minutes followed by repeated washing with autoclaved distilled water. The seeds were soaked in overnight grown broth cultures of antagonistic bacteria (log-phase containing ≈10^8^ cells mL^-1^) for 30 minutes, individually. Ten seeds were grown aseptically on moist filter paper in each Petri plate. Petri plates were incubated at a day/ night temperature of 30±2°C with the day length of 16 hours and light intensity of 20,000 Lux for 10 days. Filter papers were kept moist with autoclaved distilled water. Un-inoculated seeds were used as control. There were three replicates for each treatment. Data regarding hypocotyl and radical lengths was recorded after 10 days.

#### Experiment II: Evaluation of bacterial antagonists for disease suppression and induction of defense related enzymes

Potential of antagonistic bacteria to suppress BLB was evaluated in a pot experiment under natural environmental conditions in net house. Five bacterial antagonists E227, E233, Rh219, Rh269, Rh323 were evaluated against Xoo8. Un-inoculated plants without pathogen/ antagonistic bacteria were used as healthy control and plants inoculated with pathogen only were used as infected control. All bacterial antagonists were found compatible with each other as no clear zone of inhibition was formed in diffusion plate assay. *Bacillus* sp. Rh219 was incompatible with the rest of strains as indicated by clear zone of inhibition in compatibility test. Therefore, four strains E227, E233, Rh269 and Rh323 were used in mixed inoculum. The experiment was set up in completely randomized design (CRD) with four replicates each. Seeds of rice variety Super Basmati were dipped individually in broth cultures of antagonistic bacteria (≈10^8^ cells mL^-1^) for 45 minutes and sown directly in plastic pots of 13 cm diameter containing 1.26 Kg of autoclaved soil pot^-1^ (texture clay loam, pH 8.5, EC 4.1 mS, organic matter 0.4%, viable cell count 1.8 x 10^6^ cells g^-1^ soil). Plants were supplemented with ½ strength Hoagland solution [44] whenever required. On 21^st^ day of sowing, plants were re-inoculated by spraying with the suspension of antagonistic bacteria (10^−6^–10^−7^ cells mL^-1^) prepared in distilled autoclaved water. Control treatments were sprayed with distilled autoclaved water. Leaves of healthy control plants were clip inoculated with distilled autoclaved water while leaves of infected control (IC) plants and other inoculated treatments were clip inoculated with Xoo8 on 23^rd^ day of sowing [28]. %DLA was recorded after 21 days of clip inoculation. Plants were harvested 45 days after germination. Data regarding shoot length and plant dry weight was collected and analyzed statistically using Duncan’s Multiple Range test. Survival of antagonistic bacteria in root rhizosphere was recorded on LB medium by viable count after 14 and 21 days of sowing. Colonization potential of indigenous and inoculated bacteria was recorded. The inoculated strains were identified on the basis of their cultural characteristics and presence or absence of certain physiological characteristics as well as different extent of these traits like pattern of antagonism against different Xoo strains, different levels of P solubilization and production of siderophores and IAA.

#### Enzyme assays

Defense related enzyme assays were done 48 hours after foliar spray of antagonistic bacteria on rice plants grown under net house condition. 4–5 leaves were randomly harvested from each replicate pot containing 10 plants each. These 4–5 leaves were cut into small pieces, mixed thoroughly and 0.1g sample was processed immediately for an enzyme assay. The activity of peroxidase (POD) was assayed using guaicol as substrate following [45] and its activity was defined as the amount of enzyme that increases the absorbance at 470 nm min^-1^ [46]. The activity of Polyphenol oxidase (PPO) and catalase (CAT) was estimated following Worthington Enzyme Manual [47]. The PPO activity was determined as increase in absorbance at 280 nm and defined as that amount which produces 1mM of quinine min^-1^ [48]. The catalase activity was determined as the decrease in H_2_O_2_ at 240 nm and one unit activity equals that amount of CAT which breaks down 1μmol of H_2_O_2_ min^-1^ [49]. Phenylalanine ammonia-lyase (PAL) activity was determined by spectrophotometric measurement of the conversion of the L-phenylalanine into trans-cinnamic acid at 290 nm [45; 50; 51].

#### Experiment III: Field inoculation studies

A field experiment was carried out at NIBGE to evaluate the effect of antagonistic bacteria E227, E233, Rh269, Rh323 in combination with N_2_ fixing *Azospirillum* sp. ER20 [52]. Antagonistic strains were inoculated as mixed-culture at the time of transplantation by root dip method [53]. N and P were applied at 140 and 80 kg acre^1^ (full/ recommended NP) as urea and di-ammonium phosphate (DAP), respectively. Treatments with full/ recommended NP and with 80% of full NP without inoculation were kept as positive and negative controls, respectively. The treatments were distributed in a randomized complete block design (RCBD) with four replicates each. The plot size was 64 m^2^. DAP was added before sowing while urea was added in three split doses i.e. first one added with field preparation while 2^nd^ and 3^rd^ doses were added at 25^th^ and 50^th^ day of transplantation, respectively. Plant to plant and row to row distance was 9 inch. The plants were harvested at maturity. Plants were harvested by hand, sun dried and weighed. Grain and straw yield was recorded for the whole plot. Yield was expressed as weight of rice grain at 14% water content [54].

#### Statistical analysis

Data of pot and field experiments were analyzed statistically by analysis of variance (ANOVA) using Statistix (version 8.1) and Least significant difference (LSD) test at 5% probability. Relationship between growth parameters and %DLA was studied by correlation analysis using SPSS Software package version 17.0 (SPSS, Inc., Chicago, IL).

## Results

**Fig 1:**
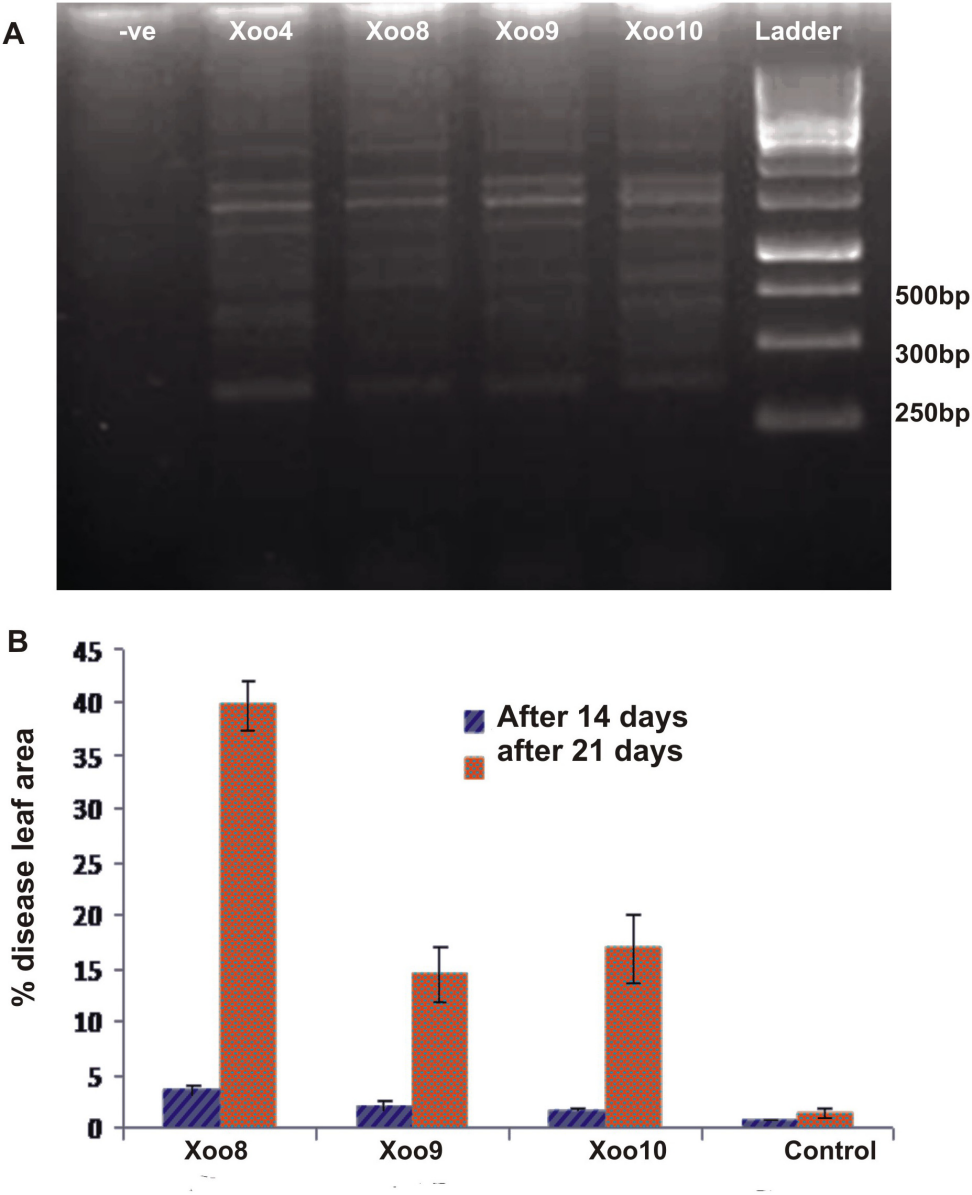
Detection of *Xanthomonas oryzae* (Xoo) pathovar (A) and confirmation of pathogenicity (B) of selected Xoo strains on rice variety Super Basmati. Xoo8, 9, 10 were isolated in the present study: Xoo4, Reference strain for pathovar detection. For pathogenicity test, control plants were clip inoculated with sterilized water. The values are an average of three replicates. Error bars show standard deviation.

**Isolation and pathogenicity test of Xoo.** Three strains Xoo8, Xoo9, Xoo10 were isolated from rice plants collected from different locations in the present study. Cell morphology was studied using light microscopy at 100X. Analysis of PCR products of Xoo strains showed the amplification of approximately 300 bp band of a molecular marker J03 specific for pathovar *oryzae*. All the Xoo strains produced typical symptoms of BLB *i*.*e*. acute wilting, yellowing of leaves starting from the tip and progressing downward on rice. Strain Xoo8 was found to be the most virulent, causing maximum % diseased leaf area (%DLA; 39.7%), followed by Xoo10 (16.8% DLA). Strain Xoo9 was least virulent strain causing only 14.3% DLA (Fig 1).

### Isolation, identification and *in vitro* screening of rhizobacteria against Xoo

**Fig 2:**
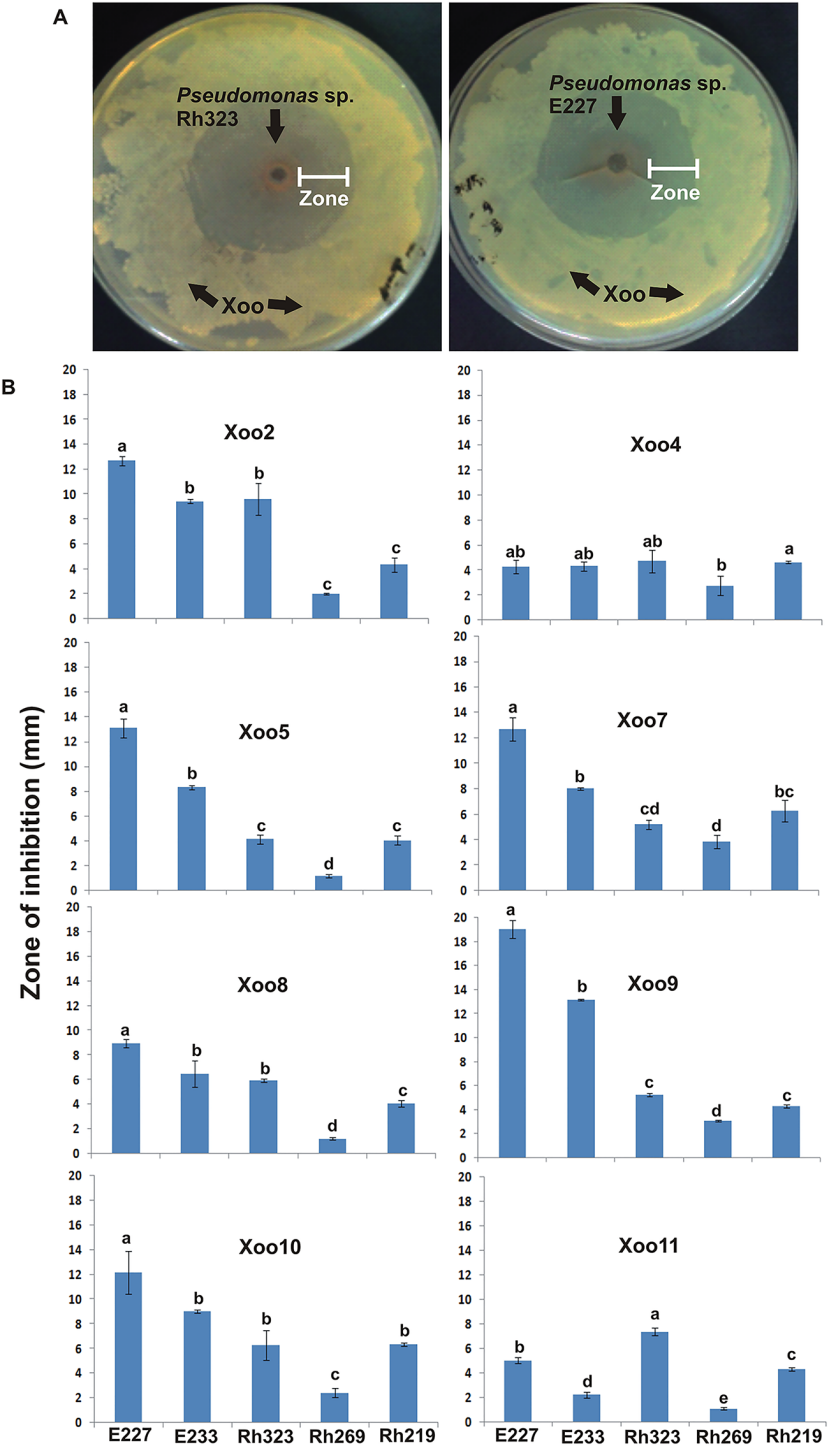
*In vitro* antagonistic activity of rhizospheric bacteria against *Xanthomonas oryzae* pv. *oryzae* (Xoo), the causal agent of Bacterial leaf blight. Zone of inhibition formed by *Pseudomonas* spp. E227 and Rh323 (A) against different strains of Xoo using hole plate diffusion method. Inhibition zones formed by *Pseudomonas* spp. E227, E233, Rh323; *Serratia* sp. Rh269; *Bacillus* sp. Rh219 (B); Values are an average of three replicates. Error bars show standard deviation. The means followed by different letters are significantly different at 1% level of significance. Xoo8, Xoo9, Xoo10 were isolated in the present study while Xoo2, Xoo4, Xoo5, Xoo7 and Xoo11 were reference strains.

A total of 811 rhizobacteria (381 rhizospheric, 233 rhizoplanic and 197 endorhizospheric) were isolated from different rice samples. Of 811 bacteria, 23 showed antagonistic activity against Xoo (Unpublished data). Among these 23 bacterial antagonists, 5 bacterial isolates E227, E233, Rh323, Rh269 and Rh219 showed *in vitro* antagonistic activity against all Xoo strains in repeated experiments. Bacterial isolate E227 showed maximum antagonistic activity against all the tested Xoo strains with zone of inhibition ranging from 1–19 mm (Fig 2). The bacterial strains were Gram negative and KOH positive except Rh219. Three bacteria E227, E233 and Rh323 gave green fluorescence at 365nm. All bacterial antagonists except E233 were positive for catalase test.

16S rRNA gene sequencing confirmed that these antagonistic bacteria belonged to the three different genera *Pseudomonas*, *Serratia* and *Bacillus*. 16S rRNA gene sequence analysis of the bacterial strain E227 showed 100% identity with *Pseudomonas aeruginosa* strain ASR-39 (accession no. KP866913.1) while rest of the 4 strains E233, Rh323, Rh 269 and Rh219 showed 99% identity with *P*. *aeruginosa* strain CD274 (Accession no. KF193637.1), *P*. *aeruginosa* (accession no. KP868686.1) *Serratia* sp. AH-6-2 (accession no. JN381538.1) and *Bacillus subtilis* strains EXWB4-09 (accession no. EU334108.1), respectively. These 16S rRNA gene sequences of bacterial strains E227 E233, Rh323 Rh269 and Rh219 were submitted to NCBI data base with accession no. HG796214, HG796216, HG796215, HG941700 and HG941699, respectively.

### Characterization of rhizobacteria for biocontrol and growth promoting traits

All bacterial strains showed the production of siderophores (Table 1). Except *Serratia* sp. Rh269, all were able to hydrolyze starch. *Pseudomonas* spp. (E227, E233 and Rh323) were positive for the production of HCN while none was positive for chitinase or protease production. *Pseudomonas* sp. E227 and *Pseudomonas* sp. Rh323 were able to produce glucanases (S1 Fig).

**Table 1:** Biocontrol and growth promoting determinants of rhizobacteria isolated from rice grown in Pakistani soils.

Antagonistic Bacteria	Biocontrol determinants				Growth promoting determinants							
	^a^Siderophores produced (mg L^-1^)	^b^ Starch hydrolysis	^c^ HCN	^d^Glucanases	^e^ IAA production(mg L^-1^)	P- Solubilization						
						^f^Solubilization index	P μg mL^-1^	^g^ Organic acids produced (μg mL^-1^)				
								AA	CA	GLA	MA	SA
*Pseudomonas* sp. E227	0.4±0.01	+++	++	+	1.85±0.1	2.6	95±3.2	0	28.0±0.4	5.6±0.2	0	0
*Pseudomonas* sp. E233	0.4±0.03	+++	++	-	0.48±0.05	2.1	82±5.3	0	10.1±0.3	0	17.5±2.1	20.1±1.8
*Pseudomonas* sp. Rh323	0.3±0.02	++	+++	+	-	2.8	98±4.5	18.5±1.6	0	2.6±0.4	4.0±0.3	0
*Serratia* sp. Rh269	0.8±0.04	-	-	-	1.23±0.07	3.5	116±6.5	5.0	13.5±0.9	0	0	28.0±2.4
*Bacillus* sp. Rh219	0.1±0.03	++	-	-	-	-	-	ND				

^a^Siderophore production was quantified using spectrophotometer.

^b^Starch hydrolysis and

^c^hydrogen cyanide (HCN) was detected by plate assay,—represents no production, ++ represents hydrolysis of starch in more than half plate, +++ represents complete hydrolysis of starch in plate.

^**d**^Glucanase production was carried out on agar medium supplemented with laminarin. + represent halo zone.

^**e**^Indole acetic acid (IAA) was quantified by HPLC.

^**f**^Solubilization index (SI) on Pikoviskaya agar; P solubilization and organic acids by antagonistic bacteria were quantified using spectrophotometer and HPLC, respectively. Acetic (AA), citric (CA), gluconic (GLA), malic (MA) and succinic (SA) acid.

^**g**^ Oxalic acid (OA) was not detected in any of the tested strains

Means are an average of three replicates, ± standard deviation

All of the tested bacteria produced IAA as indicated by pink color in spot test. HPLC analysis showed that highest amount of IAA (1.86 mg L^-1^) was produced by *Pseudomonas* sp. E227. *Pseudomonas* sp. E227, E233, Rh323 and *Serratia* sp. Rh269 formed halo zones around the bacterial growth on Pikoviskaya's agar plate after one week, indicating their potential for phosphate solubilization. *Serratia* sp. Rh269 showed highest P solubilization (116 μg mL^-1^, Table 1). *Pseudomonas* sp. Rh323 produced maximum amount of acetic acid (18.5μg mL^-1^**)** while *Pseudomonas* sp. E227 produced citric (28 μg mL^-1^) and gluconic (5.6 μg mL^-1^) acids. *Bacillus* sp. Rh233 produced maximum amount of malic acid (17.5 μg mL^-1^) and *Serratia* sp. Rh269 produced maximum amount of succinic acid (28 μg mL^-1^). Primers Phl2a/Phl2b and BPF2/BPR2, specific for 2, 4-DAPG, amplified PCR products of 746 bp and 470 bp, respectively in *Pseudomonas* sp. E227 (S2 Fig). Primers PHZ1/PHZ2 and PRND1/PRND2 specific for phenazine and pyrrolnitrin, respectively did not amplified the expected fragments from three tested *Pseudomonas* spp. strains E227, E233, Rh323.

### Plant inoculation assays

#### Effect of bacterial antagonists on seed germination

None of the selected bacterial antagonists showed any pathogenic effect on rice seedlings. Maximum hypocotyl length (3.38 cm) was recorded in seedlings inoculated with *Pseudomonas* sp. E227 while maximum radical length (2.4 cm) was observed for *Serratia* sp. Rh269 (S3 Fig).

#### *In vivo* evaluation of bacterial antagonists for disease suppression

**Fig 3:**
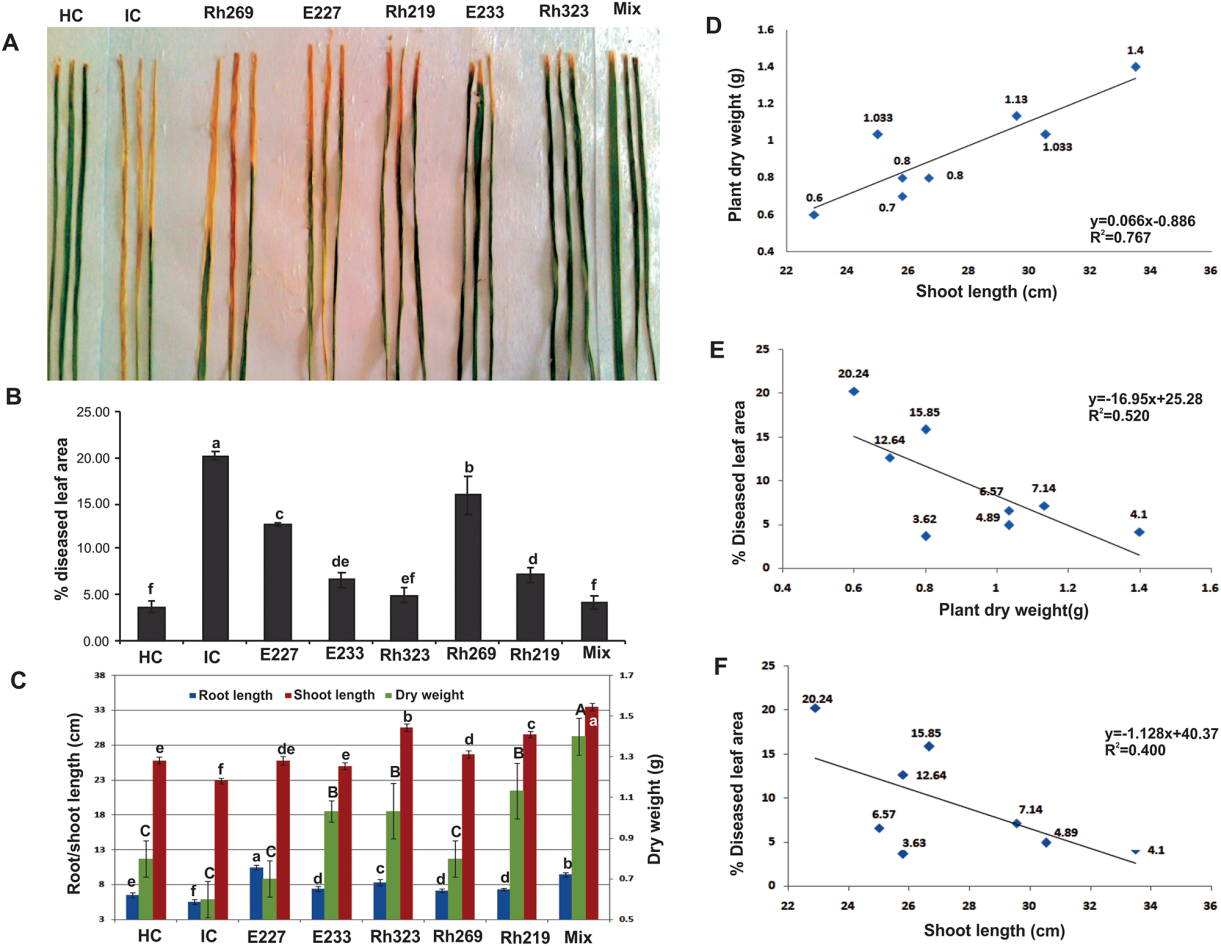
*In vivo* evaluation of antagonistic bacteria for suppression of *Xanthomonas oryzae* pv. *oryzae* (Xoo). (**A)** Clip inoculated leaves showing the severity of disease in infected control (IC) and plants inoculated with bacteria antagonists *(Pseudomonas* spp. E227, E233, Rh323; healthy control (HC) clip inoculated with distilled water showed no disease symptoms. *Serratia* sp. Rh269; *Bacillus* sp. Rh219), Mixed inoculum (E227, E233, Rh269, Rh323), (**B)** Percent Diseased leaf area (% DLA) of rice plants inoculated with bacterial antagonists and Xoo8. Values are an average of four replicates. Error bars show standard deviation of four replicates of each treatment. The means followed by different letters are significantly different at 5% level of significance. (**C**) Effect of antagonistic bacteria on shoot/ root length and plant dry weight of rice plants thirty days after seeding in net house under natural environmental conditions in the presence of Xoo8. (**D)** Relationship between plant dry weight and shoot length. (**E)** Relationship between percent diseased leaf area and plant dry weight. (**F)** Relationship between percent diseased leaf area and shoot length. Relationship between growth parameters and %DLA was studied by correlation analysis using SPSS software.

Inoculation of selected bacteria under pathogen pressure under natural environmental conditions during rice growing season (June to October) showed that the co-inoculation of strains (E227, E233, Rh269, Rh323) as well as single strain inoculum of *Pseudomonas* sp. Rh323 alone caused maximum reduction in %DLA *i*.*e*. 4.1 and 4.8, respectively as compared to that of infected control (20.2% DLA) (Fig 3A and 3B).

Plant inoculation with mixed culture and single strain inoculum of Rh323 showed a significant increase in shoot length (33.5 and 30.5 cm, respectively) as well as plant dry weight (1.0 and 0.9g, respectively) as compared to non-inoculated healthy control (Fig 3C). A positive correlation was observed between shoot length and plant dry weight at 0.05 LSD while a negative correlation was observed between %DLA and plant dry weight as well as between %DLA and shoot length at 0.05 LSD (Fig 3E and 3F).

Colonization of inoculated bacteria was detected twice after inoculation *i*.*e*., 14 and 21 days after germination. Maximum population density of indigenous soil bacteria was recorded for mixed inoculum *i*.*e*. 8.30 and 9.73 Log_10_ cfu g^-1^ soil after 14 and 21 days of germination, respectively. Density of viable population for *Serratia* sp. Rh269 was increased from 4 Log_10_ cfu g^-1^ soil to 6.62 Log_10_ cfu g^-1^ soil after 21 days of germination while *Pseudomonas* spp. E227, E233, Rh323 and *Bacillus* sp. 219 maintained their viable cells from 14 to 21 days after germination (Table 2). Red-coloured colonies of *Serratia* sp. 269 were well differentiated from the colonies of *Pseudomonas* spp. Round and flat colonies of *Pseudomonas* sp. strain Rh323 were discriminated from raised colonies of *Pseudomonas* sp. strain E227. Secondly, brown and green pigmentation produced by E233 and E277, respectively was useful for discrimination of the colonies. Antagonism and other characteristics (P solubilization, siderophores, IAA) of the inoculated bacteria were found in accordance with those as studied earlier (Table 1 and Fig 2).

**Table 2:** Population densities of indigenous and inoculated bacteria at different time intervals after germination under net house conditions.

Strains	Population density after 14 days (Log_10_ CFU g^-1^)		Population density after 21 days (Log_10_ CFU g^-1^)	
	Total viable count	Viable count of inoculated bacteria	Total viable count	Viable count of inoculated bacteria
*Pseudomonas* sp. E227	7.4±0.42	6.07±0.22	7.73±0.21	6.35±0.38
*Pseudomonas* sp. E233	7.06±0.37	5.06±0.19	7.81±0.18	6.33±0.34
*Pseudomonas* sp. Rh323	7.69±0.26	6.35±0.41	7.85±0.05	6.54±0.25
*Serratia* sp. Rh269	7.2±0.26	4.07±0.21	7.61±0.38	6.62±0.29
*Bacillus* sp. Rh219	7.36±0.3	6.29±0.46	7.50±0.41	6.30±0.31
Mixed inoculum	8.3±0.40	E227: 4.27±0.23 E233: 2.23±0.25 Rh269.0 Rh323: 3.33±0.21	9.73±0.15	E227:4.73±0.31 E233: 3.40±0.46 Rh269:3.90±0.36 Rh323: 5.20±0.36
Healthy control	7.51±0.31	0	6.80±0.20	0
Infected control	7.64±0.26	0	6.53±0.15	0

***Mixed inoculum***: *Pseudomonas* spp. E227, E233, Rh323 and *Serratia* sp. 269. Means are an average of three replicates, ± standard deviation

#### Induction of defense related enzymes

**Fig 4:**
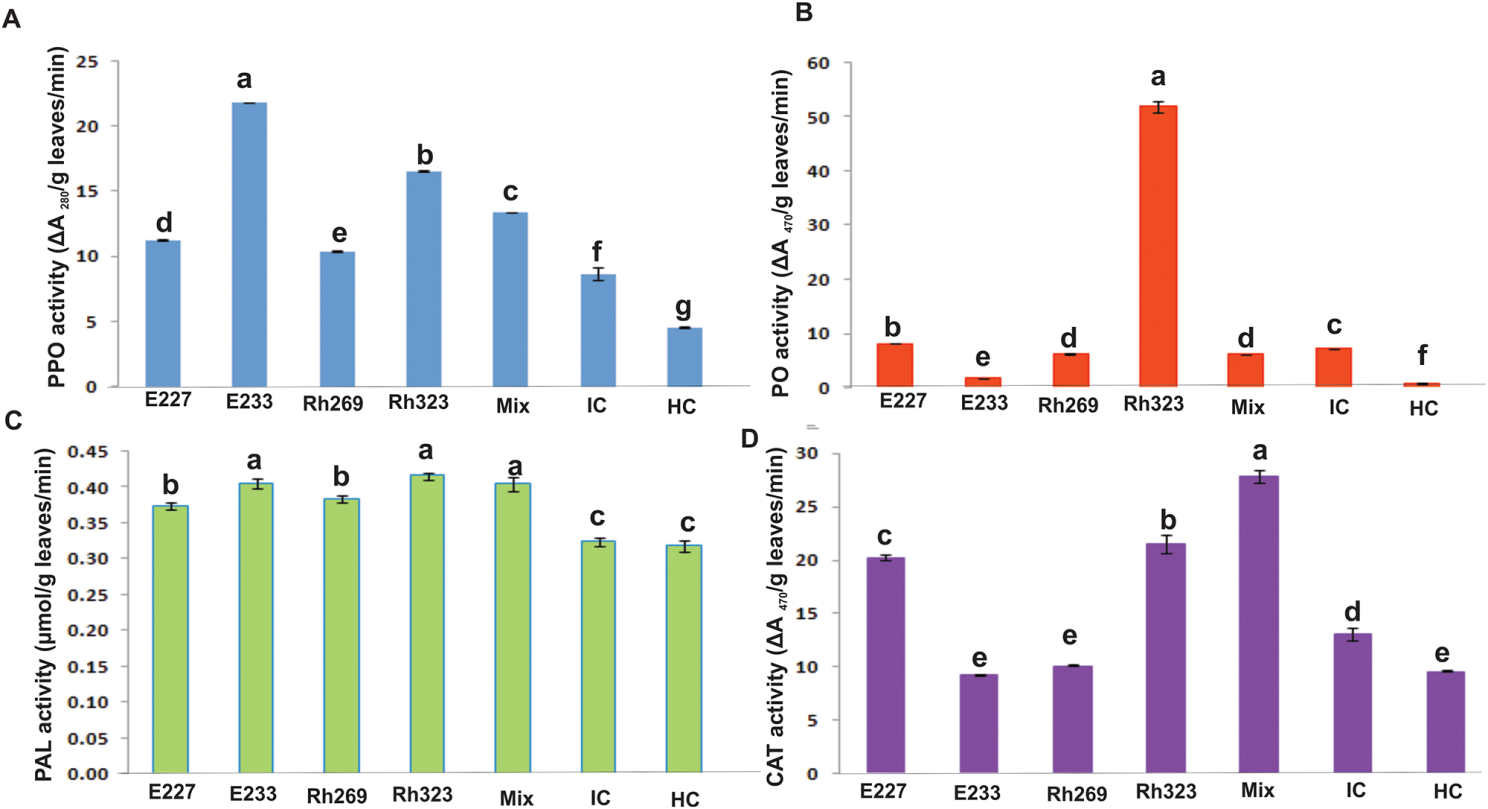
Effect of seed inoculation and foliar spray of antagonistic rhizobacteria on (A) Polyphenol oxidase (PPO), (B) Peroxidase (PO), (C) Phenylalanine ammonia-lyase (PAL) and (D) Catalase (CAT) activities in rice grown in pots under net house conditions. Foliar spray of antagonistic bacteria and clip inoculation of Xoo8 was carried out on 21^st^ and 23^rd^ day, respectively after sowing. The leaves samples were collected 48 hours after foliar spray on rice plants. Non-inoculated plants with and without pathogen, were used as infected and healthy controls, respectively. Means are an average of three replicates and bars indicate the standard deviation. Means followed by the same letter differ non-significantly at *P* = 0.05. HC: Healthy control, IC: infected control.

Polyphenol oxidase (PPO) activity was maximum in leaves of rice plants inoculated with *Pseudomonas* sp. E233 as compared to healthy and infected plants (Fig 4A). Peroxidase (PO) and Phenylalanine ammonia-lyase (PAL) activities were maximum in plants inoculated with *Pseudomonas* sp. Rh323 (Fig 4B and 4C) while CAT activity was maximum in plants treated with mixed inoculum (Fig 4D).

#### Field evaluation of bacterial antagonists for growth promotion

**Fig 5:**
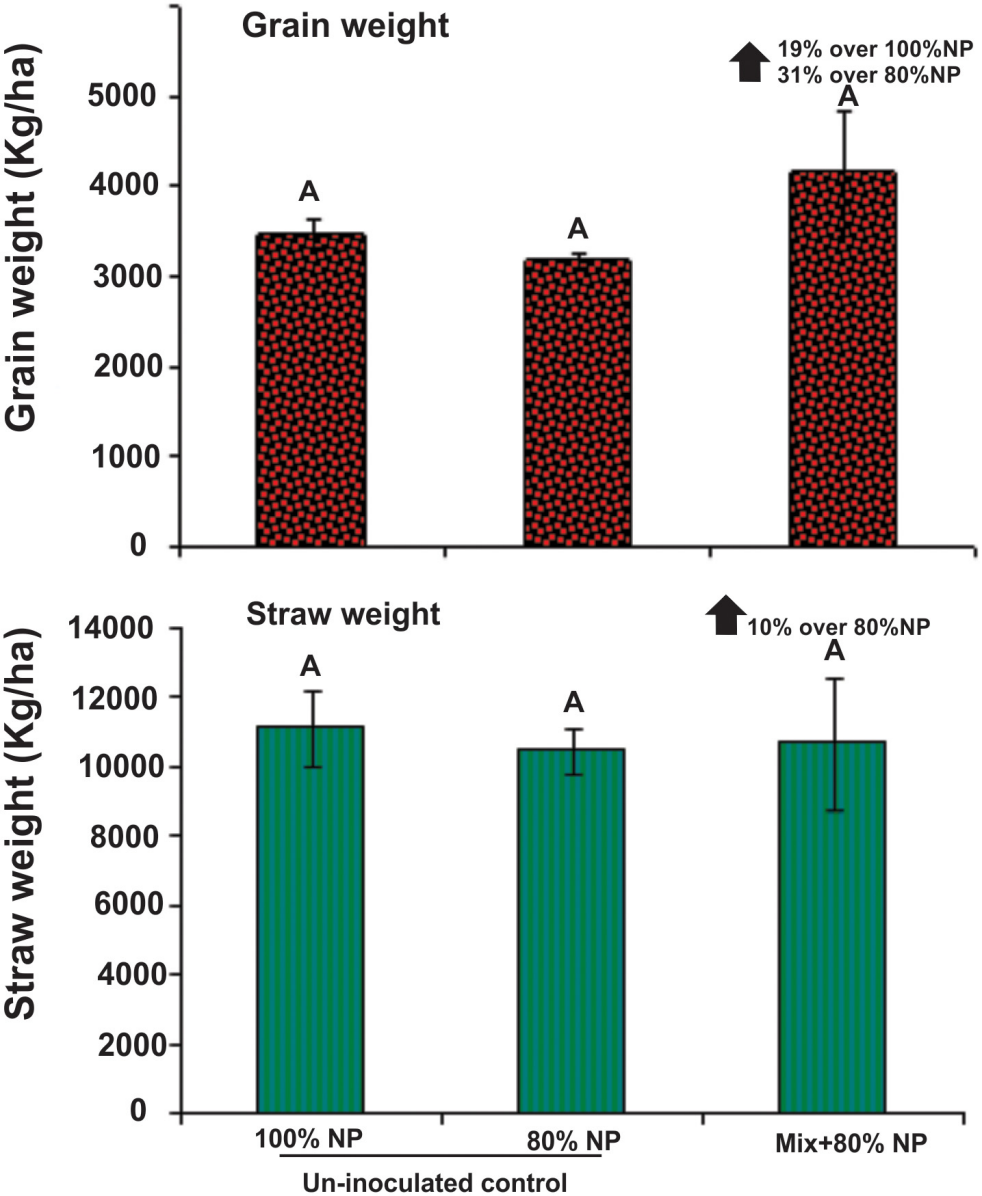
Effect of antagonistic bacteria in mixed inoculum on yield parameters of rice variety Super Basmati under field conditions. Antagonistic strains (E227, E233, Rh269, Rh323) were inoculated as mixed-culture in combination with N_2_ fixing *Azospirillum* sp. ER20 at the time of transplantation by root dip method. Treatments with full NP and with 80% of full NP without inoculation were used as positive and negative controls, respectively. Means are an average of four replicates and bars indicate the standard deviation. Means followed by the same letter differ non-significantly at *P* = 0.05.↑ shows percent increase in yield compared to the respective control.

Field trial data showed an increase of 31% in grain yield and 10% in straw yield in rice variety Super Basmati treated with mixed inoculum compared with un-inoculated control (with 80% N and P). Maximum grain yield (4166.7 kg ha^-1^) was obtained in plots treated with mixed inoculum exhibiting 19% increase over un-inoculated full-fertilized control treatment (3593.8 kg ha^-1^). Although, grain and straw yield of inoculated rice field was not statistically significant compared to its respective control plots but still % increase in yield is accomplished with reduced dose/ cost of applied chemical fertilizers (Fig 5).

## Discussion

Food security is coupled with the economic and ecological security in terms of quality and quantity of food available. Rice is an important food and export commodity of Pakistan and prone to many biotic and abiotic stresses. BLB is one of the major diseases of rice and is a main threat to rice production in both temperate and tropical regions of the world due to its high epidemic potential. Increasing demand for food and ecological security has led scientists to devise eco-friendly strategies for integrated nutrient and disease management in crops of economic importance in a more sustainable manner. Of the integrated approaches available, the use of PGPR, being ecologically safe and economically feasible, is the most attractive approach.

Present study was conducted to screen antagonistic PGPR from the rice rhizosphere for biological control of BLB and plant growth promotion.

Rice plants were collected from different sites of rice belt in Punjab province which were the known hot spots of BLB disease in the country. From disease infested plants collected from the rice growing belt, Xoo8 was found to be the most virulent strain among the prevalent pathogen strains. DNA banding pattern obtained by primer (J03) in PCR is pathovar specific and is used to identify different strains of *Xanthomonas oryzae* pv. *oryzae* on the basis of banding pattern of insertion element IS1112. Multiple bands in the gel image obtained in the present study indicated the particular pattern of IS1112 in the isolated strains of Xoo8, Xoo9 and Xoo10. Similar results have been reported in literature [55; 56].

Rhizosphere and endorhizosphere are known to be the main reservoirs of antagonistic bacteria [57], therefore, isolations of the antagonistic bacteria were carried out from both these sources. Out of 811 rhizobacteria obtained from healthy and BLB infected rice samples, five bacteria E227, E233, Rh219, Rh269, Rh323 were able to show significant and consistent antagonistic activity against all Xoo strains isolated in the present study. These potential antagonists were therefore, selected for further studies. Sequence analysis of almost full length 16S rRNA gene showed that these antagonistic bacteria belong to three different genera including *Pseudomonas* spp. strains E227 E233, Rh232, *Serratia* sp. Rh269 and *Bacillus* sp. Rh219. The PGPR and biocontrol activities of all these genera are very well known [58; 59; 60].

Biocontrol is a multidimensional and intricate phenomenon which involves several mechanisms in disease suppression. Understanding these mechanisms would be beneficial for the effective utilization of biocontrol agents in field. In the present study, all selected bacteria showed ability to produce siderophores and it is already known that production of siderophores is one of the important mechanisms involved in the suppression of BLB [61]. Furthermore, these bacteria showed the ability to hydrolyze starch, to produce cyanide, and to have glucanase activity. It has been documented earlier that microorganisms showing the ability to produce HCN can be used as biocontrol agents for the suppression of plant pathogens [62]. Glucanase activity is likely to play a role in direct antagonism [63]. PCR based detection of antibiotic-related genes indicated that *Pseudomonas* sp. strain E227 may have gene for synthesis of DAPG antibiotic. The antagonistic activity of the selected bacterial isolates might be due to the production of siderophores, lytic enzymes and HCN or synergistic interaction of these two or with other metabolites. All antagonistic strains were non-pathogenic to their host plant. Both *Pseudomonas* and *Bacillus* species are known for their broad spectrum activities against different plant pathogens [63; 64].

The antagonistic bacteria isolated in this work also showed the ability to produce phytohormone (IAA) and solubilize phosphate which shows that they can also be used for growth promotion [61]. The increased shoot length in response to bacterial inoculation (Rh323 alone as well as mixed inoculum) may be due to the effect of IAA (Fig 3). IAA is a plant growth regulating hormone that is involved in root development, elongation and proliferation thus facilitating plants to acquire more water and nutrients from soil [65]. IAA and ACC deaminase activity have been reported for plant growth promotion as well as for rhizospheric competence. Due to ethylene production under constant flooded conditions of rice, ACC deaminase activity of the inoculated bacterial strains may be of great significance [66]. Organic acid production is coupled with P solubilization in PGPR [43; 67; 68]. We found that most of the bacteria tested in this study produced citric acid whereas succinic and acetic acids were also produced in highest amount. The simultaneous release of different organic acids like acetic acid, citric acid, gluconic acid and malic acid by the rhizobacteria has been reported for their role in the solubilization of insoluble phosphates. The plant growth promotion is a complex phenomenon involving several factors e.g., root growth, nutrient acquisition, disease control and several others. Hence, the use of bacteria having multiple plant growth promoting or biocontrol properties is more feasible instead of using bacteria with single trait [69].

A significant reduction in %DLA coupled with increased shoot and root length and plant dry weight was observed in plants inoculated with mixed inoculum in pots. Significant disease reduction was observed with mixed inoculation followed by single strain inoculum of Rh323. Field evaluation further confirmed that mixed inoculum increased the yield (19%) of rice variety Super Basmati with 20% reduction in chemical fertilizers of N and P as compared to full fertilized control plots. Linear regression effectively modeled the relationship of diseased leaf area with plant dry weight and shoot length behavior at 0.05 LSD. Stimulation of root elongation in rice by PGPR inoculation has been documented [70] and is attributed to the uptake of soil nutrients [8]. The efficacy of mixed inuculum may be a synergistic effect of all bacteria and attributed to the cumulative impact of various plant growth promoting and biocontrol traits as has been reported in other studies [71].

In general, the effects of chemical fertilizers on yield parameters were higher than those of bio-inoculants alone. The use of these microbial inoculants may increase the rice yield with the saving of 20% chemical fertilizers of N and P. Assuming that the relationship between the fertilizer application and plant yield remains linear in the range 80–100% of the recommended dose, the benefit of inoculum application with reduced dose of fertilizers compared with full dose without inoculum can be substantial at country level. Adesemoye et al. [72] reported 15, 20 and 60–80% increase in the yield of wheat, rice and legume plants, respectively with the saving of 50–100% of chemical fertilizers due to microbial inoculants. Hafeez et al. [73] reported that the use of commercial biofertilizer increased the yield from 10–60% with the saving of chemical fertilizer from 30–90% for different crops. The benefit to the farmers in terms of cost per hectare was reported up to 47–426 US $.

Many factors contribute to the performance of bacterial inoculants and key amongst these is colonization. If the introduced strain does not survive substantially in the rhizosphere and colonize plant roots, it will be of no use to the plant [74]. Hence, plant colonization and rhizosphere competence are important criteria for any PGPR to be used as inoculum in field. In the present study all strains were found to colonize well in the rice rhizosphere. *Pseudomonas* spp. strains E227, Rh323 and *Bacillus* sp. Rh219 were more rhizosphere competent strains as compared to *Serratia* sp. Rh269.

The inoculated plants also showed a significant increase in the activity of defense related enzymes in rice plants. Plants inoculated with Rh323 showed a significant increase in PPO, POD and CAT activities. PPO activity was maximum in the plants treated with strain E233 while CAT activity was maximum in plants treated with mixed inoculum. The accumulation of plant defense related enzymes (POD, PPO, CAT and PAL) is related with the plant defense response and induced resistance by PGPR [46]. It is well established fact that the appropriate stimuli or signals are needed to induce defense genes [75]. Enhanced activities of defense related enzymes by PGPR inoculation has been reported in *Piper betle L*. [76] cucumber [46], wheat [77] and groundnut seedlings [78].

Furthermore, inoculation with *Pseudomonas* sp. Rh323 and mixed inoculation also showed an increase in PAL activity in treated rice plants under pathogen pressure. It is well established fact that the synthesis of phenolic compounds in plants in response to infection is associated with resistance and many studies have indicated that increased accumulation of phenolics is due to an increase in PAL activity that offers protection against diseases [79, 80].

The practicality of the whole study is that the antagonistic *Pseudomonas* spp. E227, E233, Rh323 and *Serratia* sp. Rh269 may be formulated for field application with a purpose to control BLB in conjunction with rice growth promotion. Although, *Pseudomonas* and *Serratia* spp. are wide spread and omnipresent in a variety of rhizomicrobioms but a few of them are opportunistic pathogens, hence appropriate regulation is suggested for the practical implication of this technology in the field.

## Conclusions

Plant disease suppression and growth promotion are complicated integrative phenomena that may be addressed by plant augmentation using biocontrol agents having plant growth promoting traits. As rice is an important food and export commodity, per acre yield and quality leaves a great deal to be desired. Having this in mind, we have screened a large collection of bacteria to be used in integrated disease and nutrient management strategy in rice. A rhizo-competent strain *Pseudomonas* sp. Rh323 having P-solubilization and siderophores production ability showed significant *in vivo* potential to suppress BLB coupled with rice growth promotion. This strain is also associated with the induction of disease resistance in rice due to increased accumulation and activities of plant defense enzymes. Furthermore, the potential of a bacterial consortium based on three *Pseudomonas* spp. E227, E233, Rh323 and *Serratia* sp. Rh269 has also been documented *in vivo*. Based on the results of this study, we suggest that *Pseudomonas* sp. Rh323 can be applied to suppress BLB disease and growth promotion of rice in the country. However, detailed biosafety studies of these novel biocontrol agents are required to ensure their safe release in environment and commercial use.

## Supporting Information

S1 Fig Plate assays to represent biocontrol determinants of selected antagonistic bacteria. **(A)** Control plate showing no starch hydrolysis, (**B)** Starch hydrolysis by *Pseudomonas* sp. E233, (**C)** Siderophore production by *Pseudomonas* sp. Rh323 and no siderophores detected in control i.e. siderophore non-producing bacterial strain StRh2, (**D)** Siderophores produced by *Serratia* sp. Rh269 and *Pseudomonas* sp. E233 indicated by pink coloration.
(TIF)

S2 Fig Agarose gel electrophoresis for the detection of amplified products from genomic DNA of antagonistic bacteria using primers Phl2a/Phl2b and BPF2/BPR2, specific for 2, 4-DAPG. Lane Marker: 1 kb DNA ladder, -ve: Negative control, *Pseudomonas* spp. E227, E233, Rh323; *Serratia* sp. Rh269. (TIF)

S3 Fig Germination test to study the pathogenecity of bacterial antagonists to rice seedlings. The seeds were grown on moist filter paper in sterile Petri plates under controlled conditions in a growth room. *Pseudomonas* spp. strains E227, E233, Rh323; *Serratia* sp. Rh269 *Bacillus* sp. Rh219; Control: Seeds were treated with sterilized water. Values are an average of three replicates. Error bars show the standard deviation. The means followed by different letters are significantly different at 5% level of significance.
(TIF)

S1 Table Primers and thermocycler conditions used for the amplification of antibiotic genes from antagonistic bacteria. (DOCX)

## References

[1] Molina J, Sikora M, Garud N, Flowers JM, Rubinstein S, Reynolds A, et al Molecular evidence for a single evolutionary origin of domesticated rice. Proc Natl Acad Sci. U S A. 2011; 108: 8351–8356. 10.1073/pnas.1104686108 21536870 PMC3101000

[2] Das B, Sengupta S, Prasad M, Ghose TK. Genetic diversity of the conserved motifs of six bacterial leaf blight resistance genes in a set of rice landraces. BMC Genet. 2014; 15: 82 10.1186/1471-2156-15-82 25016378 PMC4105243

[3] Ji GH, Lan FW, Yue-Qiu H, Ya Peng W, Xue HB. Biological control of rice bacterial blight by *Lysobacter antibioticus* strain 13–1. Biol Control. 2008; 45: 288–296.

[4] Montano FP, Alias-Villegas C, Bellogin RA, Cerro PD, Espuny MR, Guerrero IJ, Lopez-Baena FJ, Ollero FJ, Cubo T. Plant growth promotion in cereal and leguminous agricultural important plants: From microorganism capacities to crop production. Microbiol Res. 2014; 169: 325–336. 10.1016/j.micres.2013.09.011 24144612

[5] Hallmann J, Quadt-Hallmann A, Mahaffee WF, Kloepper JW. Bacterial endophytes in agricultural crops. Can J Microbiol. 1997; 43: 895–914.

[6] Glick BR. The enhancement of plant growth by free living bacteria. Can J Microbiol. 1995; 41: 109–117.

[7] Khan M S, Zaidi A, Ahmad E. Mechanism of phosphate solubilization and physiological functions of phosphate-solubilizing microorganisms In: Khan MS, Zaidi A, Musarrat J, editors. Phosphate solubilizing microorganisms, Principles and application of microphos Technology. Springer International Publishing Switzerland P 297 (ISBN: 978-3-319-08215-8).

[8] Panhwar QA, Naher UA, Jusop S, Othman R, Latif MA, et al Biochemical and molecular characterization of potential phosphate-solubilizing bacteria in acid sulfate soils and their beneficial effects on rice growth. Herrera-Estrella L, ed. PLoS ONE. 2014; 9 (10): e97241 10.1371/journal.pone.0097241 25285745 PMC4186749

[9] Chen L, Dodd IC, Theobald JC, Belimov AA, Davies WJ. The rhizobacterium *Variovorax paradoxus* 5C-2, containing ACC deaminase, promotes growth and development of *Arabidopsis thaliana* via an ethylene-dependent pathway, J Exp Bot. 2013; 64: 1565–1573. 10.1093/jxb/ert031 23404897 PMC3617834

[10] Ahirwar N. PGPR current and future prospects for development of sustainable agriculture. J Microbiol Biotechn. 2015; 7: 96–102.

[11] Gomez LCC, Schiliro E, Valverde CA, Mercado BJ. The biocontrol endophytic bacterium *Pseudomonas fluorescens* PICF7 induces systemic defense responses in aerial tissues upon colonization of olive roots. Front Microbiol. 2014; 5: 427 10.3389/fmicb.2014.00427 25250017 PMC4155815

[12] Vanitha SC, Umesha S. *Pseudomonas fluorescen*s mediated systemic resistance in tomato is driven through an elevated synthesis of defense enzymes. Biol Plantarum. 2011; 55: 317–322.

[13] Hammond Kosack KE, Jones JD. Resistance gene-dependent plant defense responses. Plant Cell. 1996; 8: 1773 8914325 10.1105/tpc.8.10.1773 PMC161314

[14] Yoshida K, Kaothien P, Matsui T, Kawaoka A, Shinmyo A. Molecular biology and application of plant peroxidase genes. Appl Microbiol Biotechnol. 2003; 60: 665–670. 12664144 10.1007/s00253-002-1157-7

[15] Maksimov IV, Valeev AS, Cherepanova EA, Burkhanova GF. Effect of chito oligosaccharides with different degrees of acetylation on the activity of wheat pathogen-inducible anionic peroxidase. Appl Biochem Microbiol. 2014; 50: 82–87. 10.7868/s0555109913060123 25272758

[16] Choodamani MS, Hariprasad P, Sateesh MK, Umesha S. Involvement of catalase in bacterial blight disease development of rice caused by *Xanthomonas oryzae* pv. *oryzae*. Int J Pest Manag. 2009; 55: 121–127.

[17] Sofo A, Scopa A, Nuzzaci M, Vitti A. Ascorbate peroxidase and catalase activities and their genetic regulation in plants subjected to drought and salinity stresses. Int J Mol Sci 2015; 16: 13561–13578. 10.3390/ijms160613561 26075872 PMC4490509

[18] Hameed A, Iqbal N. Chemo-priming with mannose, mannitol and H_2_O_2_ mitigate drought stress in wheat. Cereal Res Commun. 2014; 42: 450–462.

[19] Jockusch H. The role of host genes, temperature and polyphenol oxidase in the necrotization of TMV infected tobacco tissue. J Phytopathol.1966; 55: 185–192.

[20] Hemm MR, Rider SD, Ogas J, Murry DJ, Chapple C. Light induces phenyl propanoid metabolism in *Arabidopsis* roots. Plant J. 2004; 38: 765–778. 15144378 10.1111/j.1365-313X.2004.02089.x

[21] Tahsili J, Sharifi M, Safaie N, Esmaeilzadeh BS, Behmanesh M. Induction of lignans and phenolic compounds in cell culture of *Linum album* by culture filtrate of *Fusarium graminearum*. J Plant Interact. 2014; 9: 412–417.

[22] Arshad HMI, Naureen S, Saleem K, Ali S, Jabeen T, Babar MM. Morphological and biochemical characterization of *Xanthomonas oryzae* pv. *oryzae* isolates collected from Punjab during 2013. Adv life Sci. 2015; 3: 125–130.

[23] Jabeen R. Medicinal plants a potent antibacterial source against bacterial leaf blight (BLB) of rice. Pak J Bot. 2011; 43: 111–118.

[24] Wartiainen I, Eriksson T, Zheng W, Rasmussen U. Variation in the active diazotrophic community in rice paddy-nifH PCR-DGGE analysis of rhizosphere and bulk soil. Appl Soil Ecol 2008; 39:65–75.

[25] Shoebitz M, Ribaudo CM, Pardo MA, Cantore ML, Ciampi L, Cura JA. Plant growth promoting properties of a strain of *Enterobacter ludwigii* isolated from *Lolium perenne* rhizosphere. Soil Biol Biochem. 2009; 41:1768–1774.

[26] Ou SH. Rice diseases 2^nd^ ed Common wealth Mycological Institute.1985 Kew Surrey, United Kingdom.

[27] Wang CL, Zhang Q, Zhou YL, Zhao BY. Genetic diversity of pathogen *Xanthomonas oryzae* pv. *oryzae* from southern regions of Yangtze river in China. Chinese J Rice Sci. 2001; 15: 131–136.

[28] Kauffman H, Reddy A, Hsieh S, Merca S. An improved technique for evaluating resistance of rice varieties to *Xanthomonas oryzae*. Plant Disease Rep. 1973; 57: 537–541.

[29] Vincent JM. A manual for the practical study of the root nodule bacteria 1970 IBP Hand book No.15. Blackwell Scientific Publications: Oxford England.

[30] Hewitt W, Theory and application of microbiological assay. Academic press, San Diego 1989 pg.39

[31] Cappuccino J G, and Sherman N. Microbiology: a laboratory manual. 2004 Pearson Education, USA.

[32] Edward A, Civitello A, Hammond HA, Caskey CT. DNA typing and genetic mapping with trimeric and tetrameric tandem repeats. Am J Hum Genet.1991; 49: 746 1897522 PMC1683171

[33] Schwyn B, Neilands J, Universal chemical assay for the detection and determination of siderophores. Anal Biochem.1987;160: 47–56. 2952030 10.1016/0003-2697(87)90612-9

[34] Оkе OL. The role of hydrocyanic acid in nutrition. World review of nutrition and dietetics. 1969; 170–198. 4313082 10.1159/000387578

[35] Marten P, Smalla K, Berg G. Genotypic and phenotypic differentiation of an antifungal biocontrol strain belonging to *Bacillus subtilis*. J Appl Microbiol. 2000; 89: 463–471. 11021578 10.1046/j.1365-2672.2000.01136.x

[36] Denizci A, Kazan D, Abeln E, Erarslan A. Newly isolated *Bacillus clausii* GMBAE 42: an alkaline protease producer capable to grow under higly alkaline conditions. J App Microbiol. 2004; 96: 320–327. 10.1046/j.1365-2672.2003.02153.x 14723693

[37] Qing F, Shiping T, Haibo L, Yong X. Production of β-1, 3-glucanase and chitinase of two biocontrol agents and their possible modes of action. Chin Sci Bull. 2002; 47: 292–296.

[38] Zhang Y, Fernando WG, de Kievit TR, Berry C, Daayf F, Paulitz TC. Detection of antibiotic-related genes from bacterial biocontrol agents with polymerase chain reaction. Can J Microbiol. 2006; 52: 476–481. 16699573 10.1139/w05-152

[39] Patten CL, Glick BR. Role of *Pseudomonas putida* indole acetic acid in development of the host plant root system. Appl Environ Microbiol. 2002; 68: 3795–3801. 12147474 10.1128/AEM.68.8.3795-3801.2002 PMC124051

[40] Tien TM, Gaskins MH, Hubbell DH. Plant growth substances produced by *Azospirillum brasilense* and their effect on the growth of pearl millet (*Pennisetum americanum L*.). Appl Environ Microbiol. 1979; 37: 1016–24. 16345372 10.1128/aem.37.5.1016-1024.1979 PMC243341

[41] Pikovskaya RI. Mobilization of phosphorus in soil in connection with vital activity of some microbial species. Microbiologiya. 1948; 17: 362–370.

[42] Murphy J, Riley J. A modified single solution method for the determination of phosphate in natural waters. Anal Chim Acta. 1962; 27: 31–36.

[43] Tahir M, Mirza MS, Zaheer A, Dimitrov MR, Smidt H, Hameed S. Isolation and identification of phosphate solubilizer *Azospirillum*, *Bacillus* and *Enterobacter* strains by 16S rRNA sequence analysis and their effect on growth of wheat (*'Triticum aestivum* L.). Aus J Crop Sci. 2013; 7: 1284–1292.

[44] Hoagland DR, Arnon DI (1950). The water culture method of growing plants without soil. California Agri Exp Stn Circ, University of California Berkley Press, CA: 347.

[45] Hammerschmidt R, Nuckles EM, Kuc J. Association of enhanced peroxidase activity with induced systemic resistance of cucumber to *Colletotrichum lagenarium*. Physiol Plant Pathol. 1982; 20: 73–82.

[46] Liang J, Tao XR, Hao ZN, Wang LP, Zhang X. Induction of resistance in cucumber against seedling damping-off by plant growth-promoting rhizobacteria (PGPR) *Bacillus megaterium* strain L8. Afr J Biotechnol. 2011; 10: 6920–6927.

[47] Worthington CC. Worthington enzyme manual: enzymes and related biochemicals. 1988 Worthington Biochemical Corporation.

[48] Shanti R, Anannya M, Parijatham K, Mala R, Sandra SH. Extraction, purification and characterization of polyphenoloxidase from peel and pulp of tomato. Int J Curr Res Acta Rev. 2014; 2: 74–82.

[49] Weisany W, Sohrabi Y, Heidari G, Siosemardeh A, Ghassemi GK. Changes in antioxidant enzymes activity and plant performance by salinity stress and zinc application in soybean (*Glycine max* L.). Plant Omics J. 2012; 5: 60–67.

[50] Zucker M. Induction of phenylalanine deaminase by light and its relation to chlorogenic acid synthesis in potato tuber tissue. Plant Physiol. 1965; 40: 779 16656157 10.1104/pp.40.5.779 PMC550380

[51] Copping LG, Menn JJ. Biopesticides: a review of their action, applications and efficacy. Pest Manag Sci. 2000; 56: 651–676.

[52] Rasul G, Mirza SM, Latif F, Malik KA. Identification of plant growth hormones produced by bacterial isolates from rice, wheat and kallar grass In: Malik KM, Mirza SM, Ladha JK, editors. Nitrogen fixation with non legumes. Kluwer Academic Publishers 1998 pp 25–37.

[53] Tariq M, Hameed S, Malik KA, Hafeez FY. Plant root associated bacteria for zinc mobilization in rice. Pak J Bot. 2007; 39: 245–253.

[54] Wimberly JE. Technical hand book for the paddy rice postharvest industry in developing countries. International Rice Research Institute, Philippine 1983 p147 (ISBN 971-104-075-1).

[55] Adhikari TB, Mew TW, Leach JE. Genotypic and pathotypic diversity in *Xanthomonas oryzae* pv. *oryzae* in Nepal. Phytopathology. 1999; 89: 687–694. 10.1094/PHYTO.1999.89.8.687 18944682

[56] Xie J, Wang X, Li F, Peng Y, Zhou G. Three new loci of insertion element IS1112 in chinese strains of *Xanthomonas oryzae* pv. *oryzae*. J. Microbiol. 2007; 45: 219–226. 17618227

[57] Yang JH, Liu HX, Zhu GM, Pan YL, Xu LP, Guo JH. Diversity analysis of antagonists from rice associated bacteria and their application in biocontrol of rice diseases. J Appl Environ Microbiol. 2008; 104: 91–104. 10.1111/j.1365-2672.2007.03534.x 17850318

[58] Fravel DR. Commercialization and implementation of biocontrol. Annu Rev Phytopathol. 2005; 43: 337–359. 16078888 10.1146/annurev.phyto.43.032904.092924

[59] Couillerot O, Prigent-Combaret C, Caballero-Mellado J, Moenne-Loccoz Y. *Pseudomonas fluorescens* and closely-related fluorescent pseudomonads as biocontrol agents of soil-borne phytopathogens. Lett Appl Microbiol. 2009; 48: 505–512. 10.1111/j.1472-765X.2009.02566.x 19291210

[60] Mehmood MA, Hafeez FY, Xiao X, Gai Y, Wang F. Molecular characterization of an antifugal chitinase from *Serratia proteamaculans* 18A1.W J Microbiol Biotechnol. 2009; 25: 1955–1961.

[61] Naureen Z, Price AH, Hafeez FY, Roberts MR. Identification of rice blast disease suppressing bacterial strains from the rhizosphere of rice grown in Pakistan. Crop Prot. 2009; 28: 1052–1060.

[62] Ramette A, Frapolli M, Defago G, Moenne LY. Phylogeny of HCN synthase encoding hcnBC genes in biocontrol fluorescent pseudomonads and its relationship with host plant species and HCN synthesis ability. Mol Plant Microbe Interact. 2003; 16: 525–535. 12795378 10.1094/MPMI.2003.16.6.525

[63] Gustavo SM. Mechanisms of biocontrol and plant growth promoting activity in soil bacterial species of *Bacillus* and *Pseudomonas*: a review. Biocontrol Sci Techn. 2012; 22: 8558–8572.

[64] Velusamy P, Immanuel JE, Samuel SG. Rhizosphere bacteria for biocontrol of bacterial blight and growth promotion of rice. Rice Sci. 2013; 20: 356–362.

[65] Yao T, Yasmin S, Hafeez FY. Potential role of rhizobacteria isolated from North-western China for enhancing wheat and oat yield. J Agri Sci. 2008; 146: 49–56.

[66] Nascimento FX, Rossi MJ, Soares CRFS, MacConkey BJ, Glick BR. New insights into 1-aminocyclopropane-1-carboxylate (ACC) deaminase phylogeny, evolution and ecological significance. PLoS One 2014; 9: e99168 10.1371/journal.pone.0099168 24905353 PMC4048297

[67] Tahir M, Mirza MS, Hameed S, Dimitrov MR, Smidt H. Cultivation-based and molecular assessment of bacterial diversity in the rhizosheath of wheat under different crop rotations. PLoS One 2015; 10: e0130030 10.1371/journal.pone.0130030 26121588 PMC4487687

[68] Hanif MK, Hameed S, Imran A, Naqqash T, Shahid M, Van Elsas JD. Isolation and characterization of a β-propeller gene containing phosphobacterium *Bacillus subtilis* strain KPS-11 for growth promotion of potato (*Solanum tuberosum* L.). Front Microbiol. 2015; 6: 583 10.3389/fmicb.2015.00583 26106383 PMC4460431

[69] Ali S, Hameed S, Imran A, Iqbal M, Lazarovitz G. Genetic, physiological and biochemical characterization of *Bacillus* sp. strain RMB7 exhibiting plant growth promoting and broad spectrum antifungal activities. Microb Cell Fact. 2014; 13: 144 10.1186/s12934-014-0144-x 25338952 PMC4213516

[70] Hafeez FY, Safdar M E, Chaudhry AU, Malik KA. Rhizobial inoculation improves seedling emergence, nutrient uptake and growth of cotton. Aust J Exp Agr. 2004; 44: 617–622.

[71] Jetiyanon K, Kloepper JW. Mixtures of plant growth promoting rhizobacteria for induction of systemic resistance against multiple plant diseases. Biol Control. 2002; 24: 285–291.

[72] Adesemoye AO, Torbert HA, Kloepper JW. Plant growth-promoting rhizobacteria allow reduced application rates of chemical fertilizers. Microb Ecol. 2009; 58: 921–929. 10.1007/s00248-009-9531-y 19466478

[73] Hafeez FY. 2009. Benefits of a tiny creature, a success story of biofertilizer technology in Pakistan. In: Hafeez FY., Malik, KA. & Zafar Y, editors. Microbial technologies for sustainable agriculture, Exploring the hidden potentials of microbes. Proceeding of the international symposium on microbial technologies for sustainable agriculture from 12–16 March, 2007, Faisalabad, Pakistan. pp. 279-282. ISBN: 978-969-8189-14-3.

[74] Hiddink GA, Termorshuizen AJ, Raaijmakers JM, Van Bruggen AH. Effect of mixed and single crops on disease suppressiveness of soils. Phytopathology 2005; 95: 1325–1332. 10.1094/PHYTO-95-1325 18943364

[75] Saikia R, Yadav M, Varghese S, Singh BP, Gogoi DK, Kumar R, et al Role of riboflavin in induced resistance against *Fusarium* wilt and charcoal rot diseases of chickpea. Plant Pathol J. 2006; 24: 339–347.

[76] Lavania M, Chauhan PS, Chauhan SVS, Singh HB, Nautiyal CS. Induction of plant defence enzymes and phenolics by treatment with plant growth promoting rhizobacteria *Serratia marcescens* NBRI 1213. Curr Microbiol. 2006; 52: 363–368. 16586018 10.1007/s00284-005-5578-2

[77] Hassan T, Bano A. The stimulatory effects of L-tryptophan and plant growth promoting rhizobacteria (PGPR) on soil health and physiology of wheat. J Soil Sci Plant Nutri. 2015; 15:190–201.

[78] Mathivanan S, Chidambaram A, Sundaramoorthy P, Baskaran L, Kalaikandhan R. The Effect of plant growth promoting rhizobacteria on groundnut (*Arachis hypogaea* L.) seed germination and biochemical constituents, Int J Curr Res Aca Rev. 2014; 2:187–194.

[79] Schiliro E, Ferrara M, Nigro F, Mercado-Blanco J. Genetic responses induced in olive roots upon colonization by the biocontrol endophytic bacterium *Pseudomonas fluorescens* PICF7. PLoS One. 2012; 7: e48646 10.1371/journal.pone.0048646 23144916 PMC3492495

[80] Jayaraj J, Yi H, Liang GH, Muthukrishnan S, Velazhahan R. Foliar application of *Bacillus subtilis* AUBS1 reduces sheath blight and triggers defense mechanisms in rice. J Plant Dis Protect. 2004; 111: 115–125.

