# Supplementary figures and images for: Plant Growth Promotion and Suppression of Bacterial Leaf Blight in Rice by Inoculated Bacteria
Source: PLoS One. 2016 Aug 17;11(8):e0160688. doi: 10.1371/journal.pone.0160688 (PMC4988697; doi:10.1371/journal.pone.0160688)

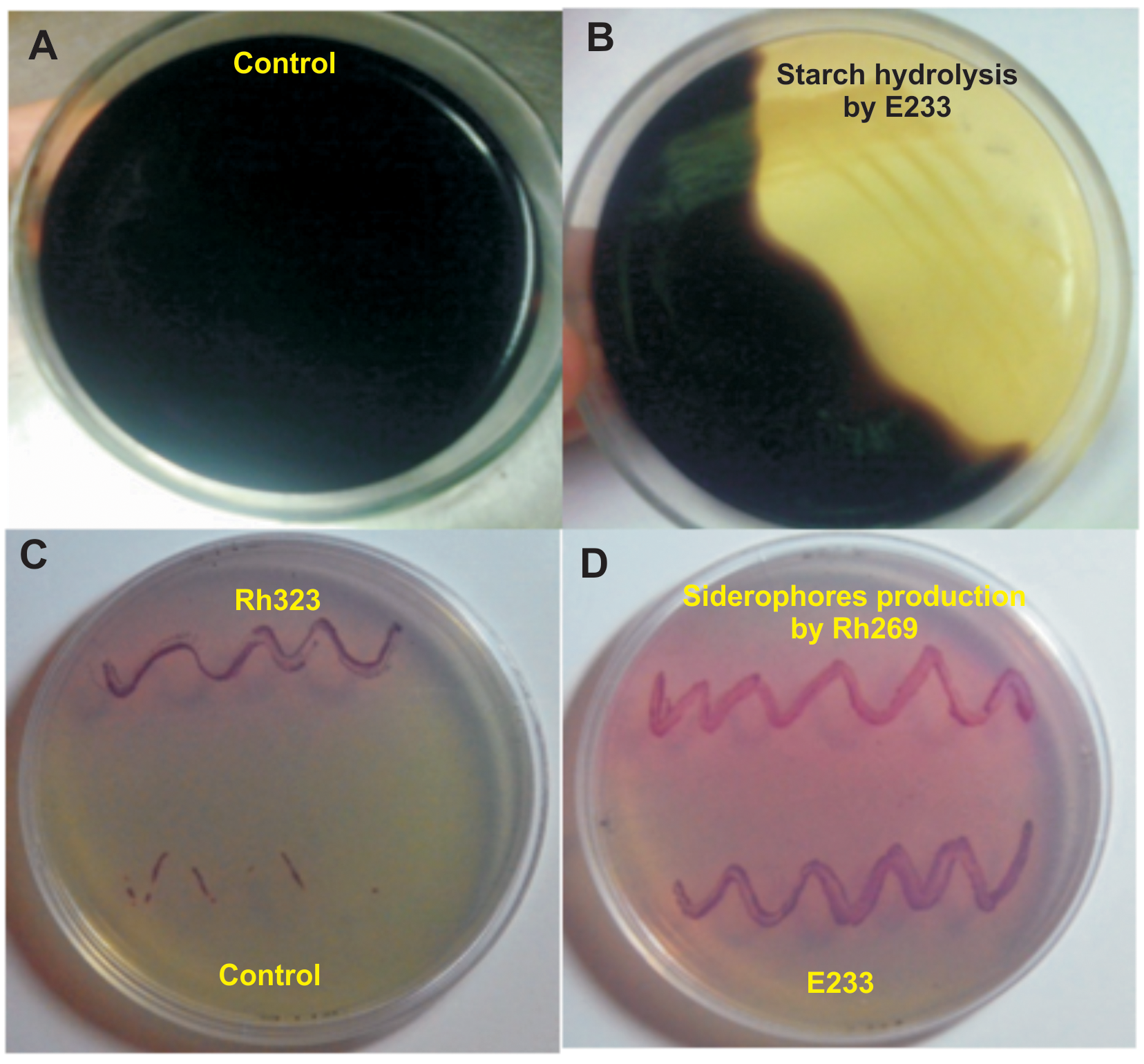

Supplement: S1 Fig — (A) Control plate showing no starch hydrolysis, (B) Starch hydrolysis by Pseudomonas sp. E233, (C) Siderophore production by Pseudomonas sp. Rh323 and no siderophores detected in control i.e. siderophore non-producing bacterial strain StRh2, (D) Siderophores produced by Serratia sp. Rh269 and Pseudomonas sp. E233 indicated by pink coloration. (TIF) [file pone.0160688.s001.tif]

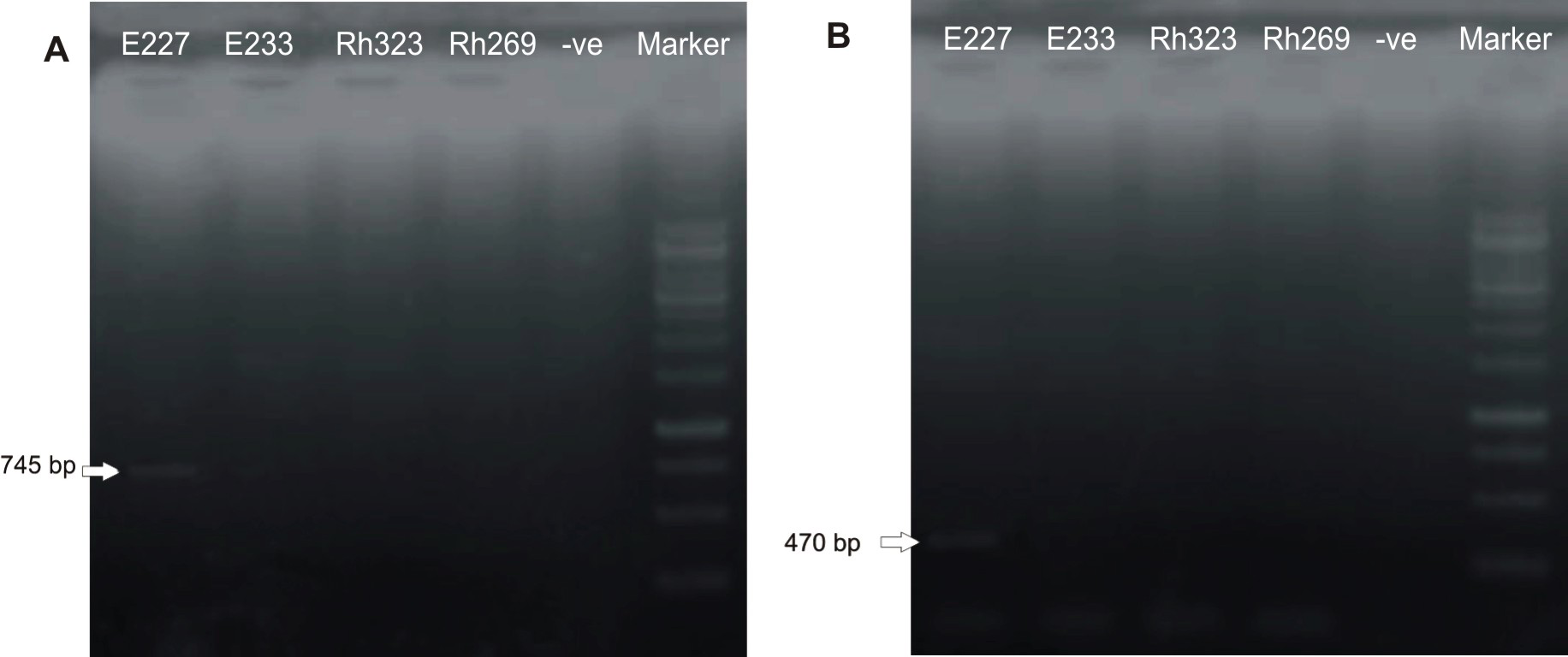

Supplement: S2 Fig — (TIF) [file pone.0160688.s002.tif]

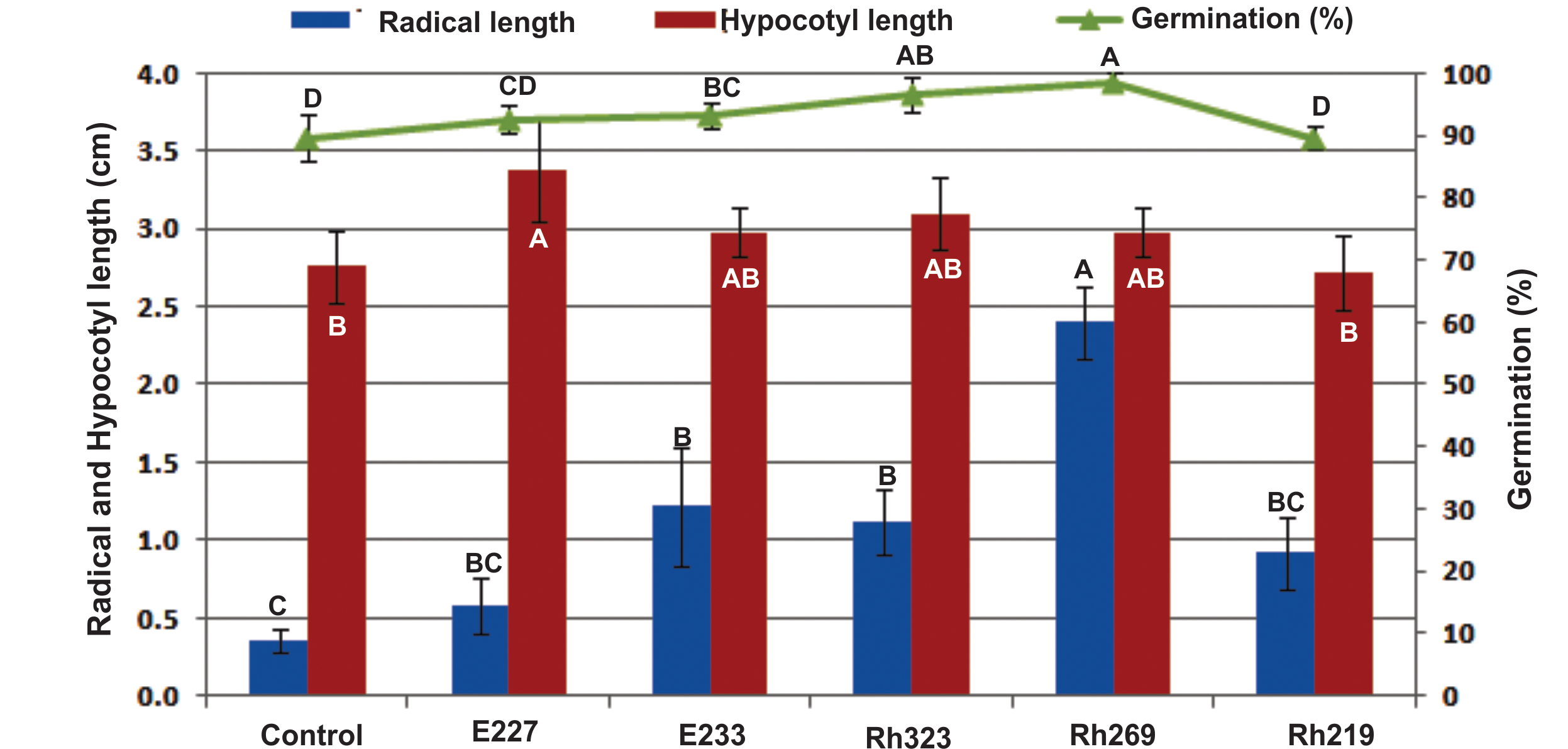

Supplement: S3 Fig — The seeds were grown on moist filter paper in sterile Petri plates under controlled conditions in a growth room. Pseudomonas spp. strains E227, E233, Rh323; Serratia sp. Rh269 Bacillus sp. Rh219; Control: Seeds were treated with sterilized water. Values are an average of three replicates. Error bars show the standard deviation. The means followed by different letters are significantly different at 5% level of significance. (TIF) [file pone.0160688.s003.tif]
