# Supplementary material for: Plant Growth Promotion and Suppression of Bacterial Leaf Blight in Rice by Inoculated Bacteria
Source: PLoS One. 2016 Aug 17;11(8):e0160688. doi: 10.1371/journal.pone.0160688 (PMC4988697; doi:10.1371/journal.pone.0160688)
Supplement: S1 Table — (DOCX) [file pone.0160688.s004.docx]

**S1 Table. Primers and thermocycler conditions used for the amplification of antibiotic genes from antagonistic bacteria**

| **Antibiotic primers** | **Target gene /primer sequence 5'-3'** | **Gene (size)** | **PCR profile** | **Reference** |
| --- | --- | --- | --- | --- |
| PHZ1  PHZ2 | **Phenazine**  GGCGACATGGTCAACGG  CGGCTGGCGGCGTATAT | phzFA/CD  (1.4kb) | Denaturation: 94°C for 1 min.  Annealing: 56°C for 1 min.  Extension: 72°C for 1.75 min. | Delany et al., 2001 |
| Phl2a  Phl2b | **2-4-Diacetylphloroglucinol**  GAGGACGTCGAAGACCACCA  ACCGCAGCATCGTGTATGAG | phlD  (746bp) | Denaturation: 94°C for 1 min.  Annealing: 67°C for 1 min.  Extension: 72°C for 1min. | Rajjimaker et al., 1997 |
| BPF2  BPR2 | **2-4-Diacetylphloroglucinol**  ACATCGTGCACCGGTTTCATGATG  GAGCGCAATGTTGATTGAAGGTCTC | phlD  (470bp) | Denaturation: 94°C for 1 min.  Annealing: 60°C for 1 min.  Extension: 72°C for 1 min. | McSpadden Gardener et al., 2001 |
| PRND1  PRND2 | **Pyrrolnitrin**  GGGGCGGGCCGTGGTGATGGA  YCCCGCSGCCTGYCTGGTCTG | prnD  (790bp) | Denaturation: 94°C for 1 min. Annealing: 68°C for 1 min.  Extension: 72°C for 90 sec. | deSouza and Rajjimaker, 2003 |

Initial denaturation: 95°C for 2 minutes; Final extension: 72°C for 5 minutes; PCR cycles: 40

Total genomic DNA was extracted by CTAB method (Ausubel et. al. 1995). The PCR reactions were performed in 50 µL reaction mixture containing 10X PCR buffer (Invitrogen); 25 mM MgCl_2_ (Invitrogen); BSA (Bovine Serum Albumin); 4mM dNTPs (Invitrogen); 10 µM forward primer; 10 µM reverse primer; 0.5 U Taq polymerase (Invitrogen) and 50 ng DNA template.

**References:** Ausubel FM, Brent R, Kingston RE, Moore DD, Seidman JG, Smith JA, Struhl K, Albright LM, Coen DM, Varki A. Current protocols in molecular biology. New York: John Wiley & Sons; 1995.

Delany SM, Mavrodi D, Bonsall VRF, Thomashow LS. 2001. phzO, a gene for biosynthesis of 2-hydroxylated phenazine compounds in Pseudomonas aureofaciens 30-84. J. Bacteriology 183: 318-327.

Rajjinmaker JM, Weller DM, Thomashow LS. Frequency of antibiotic-producing Pseudomonas spp. in natural environments. Applied Environ Microbiol. 1997; 63: 881-887.

McSpadden Gardener BB, Mavrodi DV, Thomashow LS, Weller DM. A rapid polymerase chain reaction-based assay characterizing rhizosphere population of 2, 4-diacetylphloroglucinol-producing bacteria. Phytopathology 2001;, 91: 44-54.

De Souza JT, and Rajjimaker JM, 2003. Polymorphisims within the prnD and phC genes from pyrrolnitrin and pyoluteorin-producing Pseudomonas and Burkholderia spp. FEMS Micobiol Ecol. 43: 21-34.
